# Upscaling Thermoelectrics: Micron-Thick, Half-a-Meter-Long Carbon Nanotube Films with Monolithic Integration of p- and n-Legs

**DOI:** 10.1021/acsaelm.3c01671

**Published:** 2024-03-05

**Authors:** Osnat Zapata-Arteaga, Bernhard Dörling, Ivan Alvarez-Corzo, Kai Xu, Juan Sebastián Reparaz, Mariano Campoy-Quiles

**Affiliations:** Instituto de Ciencia de Materiales de Barcelona (ICMAB-CSIC), Bellaterra 01893, Spain

**Keywords:** organic thermoelectrics, CNT, processing, large scale, thermoelectric generator, doping

## Abstract

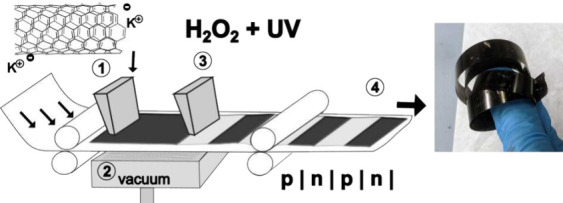

In order for organic thermoelectrics to successfully establish their own niche as energy-harvesting materials, they must reach several crucial milestones, including high performance, long-term stability, and scalability. Performance and stability are currently being actively studied, whereas demonstrations of large-scale compatibility are far more limited and for carbon nanotubes (CNTs) are still missing. The scalability challenge includes material-related economic considerations as well as the availability of fast deposition methods that produce large-scale films that simultaneously satisfy the thickness constraints required for thermoelectric modules. Here we report on true solutions of CNTs that form gels upon air exposure, which can then be dried into micron-thick films. The CNT ink can be extruded using a slot-shaped nozzle into a continuous film (more than half a meter in the present paper) and patterned into alternating n- and p-type components, which are then folded to obtain the finished thermoelectric module. Starting from a given n-type film, differentiation between the n and p components is achieved by a simple postprocessing step that involves a partial oxidation reaction and neutralization of the dopant. The presented method allows the thermoelectric legs to seamlessly interconnect along the continuous film, thus avoiding the need for metal electrodes, and, most importantly, it is compatible with large-scale printing processes. The resulting thermoelectric legs retain 80% of their power factor after 100 days in air and about 30% after 300 days. Using the proposed methodology, we fabricate two thermoelectric modules of 4 and 10 legs that can produce maximum power outputs of 1 and 2.4 μW, respectively, at a temperature difference Δ*T* of 46 K.

## Introduction

Organic thermoelectrics have emerged as a promising and rapidly evolving field in the realm of energy harvesting and conversion, utilizing organic semiconductors (OSCs) to capture waste heat and convert it to usable electricity. While it is true that organic thermoelectric materials currently exhibit a performance that is generally lower than that of their inorganic counterparts, they are often touted for other competitive advantages like abundance, ease of processability, favorable mechanical properties, and comparatively low toxicity.^1^ However, for organic thermoelectrics to establish their own niche and succeed, they must achieve significant milestones, including (i) high performance, (ii) long-term stability, and (iii) scalable manufacturing.

In terms of performance, an efficient thermoelectric material combines high electrical conductivity (σ), a high Seebeck coefficient (*S*), and low thermal conductivity (κ). These parameters are material properties that comprise the dimensionless thermoelectric figure of merit, *zT* = *S*^2^σκ^–1^*T*. Most inorganic-based thermoelectrics in the market exhibit *zT* ≈ 0.7, while only a few demonstrations in the range of 0.1–0.4 are available for their organic counterparts.^2−4^ Perhaps the most relevant difference between organic and inorganic materials from the perspective of thermoelectric conversion is their substantially different electrical and thermal conductivities. Still, the magnitude of these properties will depend on the target application, e.g., low κ for thermoelectric applications, whereas a well-defined range of thermal conductivity values is required for Peltier cooling applications.

For example, CNTs exhibit electrical conductivities that range from 500 to 10000 Ω^–1^ cm^–1^,^5−7^ with thermal conductivities between 20 and 700 W m^–1^ K^–1^.^5,8^ On the other hand, conjugated polymers (CPs) typically exhibit electrical conductivities ranging from 0.01 to 1000 Ω^–1^ cm^–1^,^9,10^ with thermal conductivities that are generally an order of magnitude lower, i.e., 0.1–1 W m^–1^ K^–1^.^11^ Hence, significant efforts from several groups are aimed at improving the doping and processing methods of OSCs to enhance their thermoelectric performance (*zT*).^4,10,12^ Regarding their stability, thermoelectric generators require both p- and n-type OSC materials. However, achieving high-performance n-type OSCs is challenging due to their susceptibility to oxidation and side reactions upon exposure to air. Progress on this front includes fine-tuning the size of the dopant and rational design of the polymer and dopants to increase the interaction between them or limit the mobility of the dopant within the OSC.^6,13−16^ Only very recently, stable n-type thermoelectrics based on OSCs have been discovered through a combination of oxidative polymerization and *in situ* reductive n doping.^17^ Finally, when considering the scalability of organic thermoelectrics, most CPs studied for thermoelectric applications are solution-processable and, hence, compatible with large-scale fabrication processes like slot-die coating, roll-to-roll processing, or inkjet printing. In fact, there are already demonstrations of CP-based thermoelectric generators fabricated with large-scale processing methods, many of them involving poly(3,4-ethylenedioxythiophene) derivatives.^7,18−21^ By contrast, CNTs are not soluble in organic solvents and, in their pure form, can thus only be crudely dispersed.^22−25^ Alternative options to improve the processability of CNTs include the use of surfactants or mixtures with polymers/binders to form a composite.^24,26−32^ The processability of these composites/dispersions will vary depending on the additive (surfactant, polymer, or binder) and so will their thermoelectric properties due to a “diluting” effect of the active material within the additive.^30,33,34^ While the binder/surfactant additive can provide added benefits (e.g., mechanical)^26^ it may be removed in postprocessing when its only purpose is that of imparting processability^35^ Still, most demonstrations so far lack the required thickness to prepare low-resistance modules.^36^

Typical methods to produce CNT films include spray coating,^16,22,23,29^ filtering,^5,28,37−39^ and wet spinning.^40,41^ While spray coating is a scalable method,^22,23^ for instance, used in the car industry, the low solubility of CNTs in most solvents implies that typically spray-coated CNT films are very thin (tens of nanometers), and only a few representative examples of micron-thick layers exist;^22,23^ thus, in most scenarios, it is considered a rather slow fabrication process. Filtering is normally considered to be a batch-to-batch process in which buckypaper is fabricated. It can produce relatively thick films, provided that a large volume of the solution is filtered. A natural thickness maximum is achievable with this method, arising from the increased difficulty of the solution to cross the CNT mats. Despite these limitations, there are some elegant examples that employ filtration and spray-coating methodologies to fabricate CNT films with large areas,^29,33,37^ and a new approach has even been proposed to combine filtration with a roll-to-roll approach.^42,43^ However, this method is still very slow due to the need to filter large volumes of a CNT dispersion through the filter substrate. As a consequence, an example of truly processable and large-scale compatible CNT-based inks for the fabrication of organic thermoelectric modules is overdue.

This study presents a method for preparing carbon nanotube (CNT)-based organic thermoelectric generators (OTEGs) using a roll-to-roll-compatible fabrication approach. First, a CNT solution with a rather high concentration of CNTs is employed, enabling the efficient and rapid production of micron-thick films. Second, the same CNT ink is used for the fabrication of the n- and p-type components of the thermoelectric module, as well as for electrode components through a postprocessing step involving partial oxidation of the CNTs and neutralization of the dopant. Finally, we demonstrate that it is possible to integrate the resulting film within the OTEG module via a simple folding process.

## Experimental Section

### Solutions

A stock solution of reductive potassium naphthalenide (K-Np) was prepared by mixing 0.83 mmol of potassium (Merck, Sigma-Aldrich) and 1 mmol of naphthalene (Merck, Sigma-Aldrich) in 10 mL of dimethylacetamide (DMAc) and stirring for 3 h using a glass-covered magnetic bar. Single-walled carbon nanotubes, either eDIPS (Meijo Nano Carbon) or CoMoCAT [Merck, Sigma-Aldrich, (6,5) chirality], were dried inside a reactor beaker at 400 °C for 3 h under vacuum and then cooled for further use. The CNT salts of different carbon-to-potassium ratios were prepared by mixing dry CNTs with the appropriate K-Np stock solution and diluting if necessary to reach a target ratio and concentration (between 0.3 and 0.5 mg mL^–1^ for this work). All solutions were prepared in a glovebox and stirred for at least 3 days before use. For the solutions prepared in dimethyl sulfoxide (DMSO), first, the K-Np stock solution was prepared in tetrahydrofuran (THF). Different carbon-to-potassium ratios were prepared by mixing dry CNTs with stock K-Np. Then, the CNT dispersion was filtered, washed with a copious amount of THF, and dried inside the glovebox. Then, CNTs were dissolved in an appropriate amount of DMSO to reach a target concentration.

### Film and Device Fabrication

CNT films were fabricated outside the glovebox to allow the formation of gel-like structures, which were then dried and transferred to a target substrate. Because we did not have any roll-to-roll equipment on hand, we used a Büchner funnel as a first embodiment, which was passed along the underside of the substrate manually, as visualized in Figure S1. For this, 10 mL of the CNT-salt solution was deposited with a syringe through a custom 3D-printed slot die onto a poly(tetrafluoroethylene) (PTFE) filter sheet (1 μm pore size, BOLA). Then, the CNT layer was dried piecewise by sliding the PTFE sheet over the Büchner funnel. The CNT films were then transferred to a poly(ethylene terephthalate) (PET) substrate and dried under vacuum at 100 °C for 1 h. Using this approach, we were able to fabricate films with thicknesses between 400 and 1000 nm, depending on the solution concentration and amount of solution used. Figures S1–S3 show photographs of the fabrication process. Single n-type legs were cut into 1 cm × 1 cm squares and contacted with silver paint. p-type legs were prepared by converting n-type sections of the film. This was achieved by immersing the films in hydrogen peroxide (H_2_O_2_) and placing them under a 250 nm, 60 W UV lamp for 20 min. Consecutive n/p legs for the OTEGs were fabricated from single 5–10 cm × 1 cm CNT films, which were dedoped/patterned in the same way. Before dedoping, designated n-type legs were protected with a spray-coated layer of PCB lacquer (RS Components Ltd.) applied through a shadow mask. Then, the film was folded into a finished module. Finished modules were clamped between two corks to provide better mechanical rigidity. Additionally, to improve the heat transfer between the module and the heat source/sink, the sides of the thermoelectric module were connected to a copper sheet using a nonelectrically conductive thermal paste.

### Infrared and Microscopy

Fourier transform infrared (FTIR) absorption spectra were measured by using a Bruker HYPERION microscope coupled to a VERTEX 70 spectrometer. All measurements were done on free-standing films attached to a poly(methyl methacrylate) holder with a 3-mm-diameter window. Scanning electron microscopy (SEM) was measured using a QUANTA FEI 200 FEG-ESEM microscope and transmission electron microscopy (TEM) with a JEOL 1210 microscope at the Institute of Material Science of Barcelona.

### Performance Measurements

#### Thermal Conductivity

The in-plane thermal diffusivity was obtained by a recently developed method providing enhanced sensitivity to in-plane thermal transport and named after “beam-offset frequency-domain thermoreflectance”.^44,45^ All measurements on the CNT-suspended films were done in vacuum conditions with a base pressure *P* < 10^–4^ mbar. This method employs a harmonically modulated (amplitude) line-shaped focused laser as the heat source, whereas the probe beam was focused to a pointlike Gaussian spot. The phase lag between the pump (heater) and the probe beams (thermometer) was measured as a function of their spatial offset, hence obtaining the in-plane thermal diffusivity of the samples. The thermal conductivity was obtained using the following relationship:
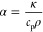
1where α is the thermal diffusivity, κ is the thermal conductivity, *c*_p_ is the heat capacity, and ρ is the density. The values of *c*_p_ and ρ were taken from previous determinations.^8,46^ Additional information and representative measurements are shown in Figure S7.

#### Seebeck Coefficient and Electrical Conductivity

Single films were measured in-plane using a custom-built setup described in a previous publication.^47^ The films were cut into 1 cm × cm squares and contacted at the corners using silver paint. The electrical conductivity was measured using the van der Pauw method, while the Seebeck coefficient was determined by heating one side of the sample and the Seebeck voltage was measured at opposite ends.

#### TEG Modules

The modules were characterized by measuring their resistance and Seebeck coefficient in a similar setup that has been adapted for the typical out-of-plane geometry of thermoelectric generators. The OTEGs were sandwiched between two aluminum blocks above and below, which served as a heater and a heat sink, respectively. Then, for a constant temperature difference, the voltage drop across a load resistor was measured for resistor values ranging from a short circuit (≈1 Ω) to an open circuit (1 MΩ). This set of measurements was then repeated for several more applied temperature differences. The current and power were then calculated from the measured voltages and known load resistances. A typical measurement of the Seebeck coefficient takes about 1 h, during which one side of the sample is exposed to 70 °C.

## Results and Discussion

**Figure 1:**
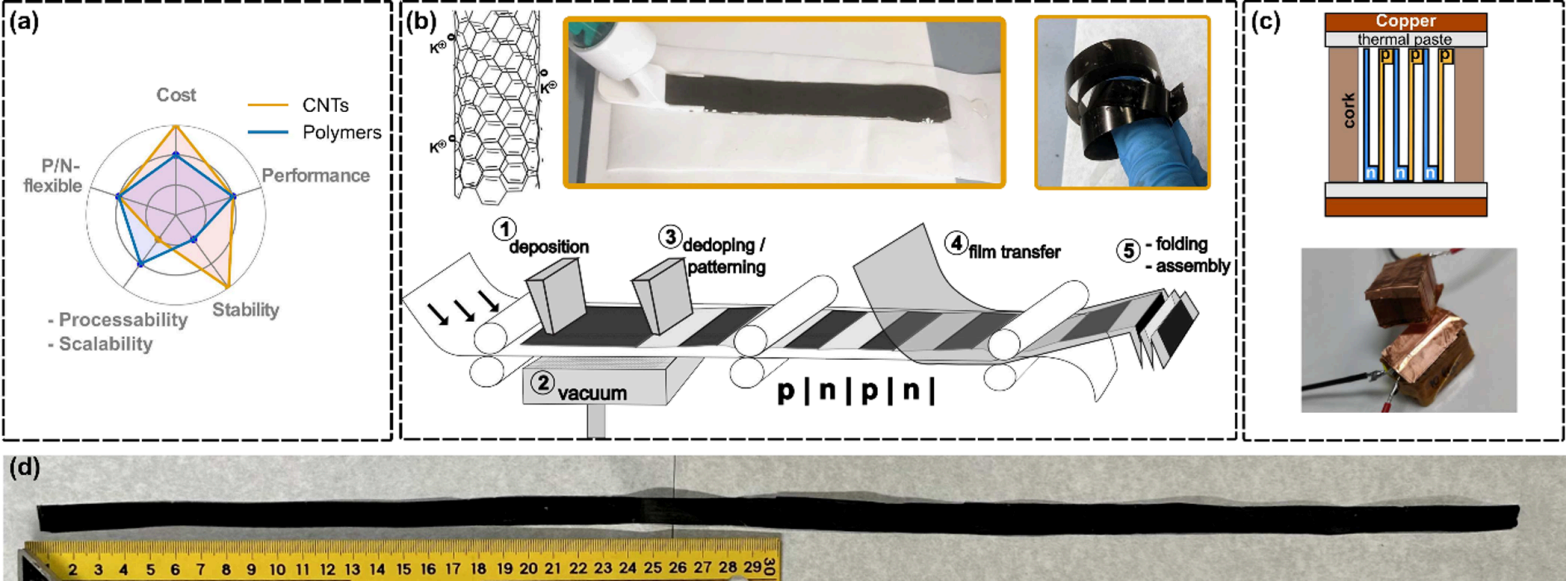
(a) Relevant properties of CNTs and CPs for the fabrication of large-scale OTEGs. (b) Schematic representation of the fabrication process described in this work. The inset photograph shows a 1-cm-thick gel, which forms after deposition. (c) Cross section of a folded film and photograph of a finished module. (d) Photograph of a ≈60-cm-long CNT film fabricated with the method depicted in part b.

Given their exceptional properties, we focus on the thermoelectric performances of CPs and CNTs, which have already been extensively discussed in recent publications.^2,4,11,31,32,37,48−52^Figure 1a provides a qualitative summary of their relative advantages and drawbacks. We focus on the use of CNTs as we aim to exploit our recent developments in their processability, which we aim to adapt to available large-scale printing techniques. Initially, we approached the problem by using a suitable method that allowed us to prepare n-type-doped buckypapers composed of CNTs with relatively small bundle diameters.^5^ Although the obtained films have shown to be promising for our main purposes, the CNT solutions used to prepare them are only stable under an inert atmosphere due to the solubilization process involving alkali metals. Upon exposure to air, the CNTs quickly aggregate in solution, forming a gel-like structure of entangled CNTs and trapped solvent, as shown in the inset of Figure 1b. To our knowledge, this effect is due to the partial formation of oxygen functionalities that occur in the presence of moisture and oxygen^53^ and does not occur with the aid of freeze-drying or supercritical-drying methodologies.^50^ This effect occurs at concentrations above 0.3 mg mL^–1^ for CNT sources with lengths between 5 and 15 μm (according to the manufacturer, eDIPS Meijo) and at concentrations close to 1 mg mL^–1^ for shorter CNT sources like CoMoCAT (1.5 μm median length according to the manufacturer).

To remove the solvent and collapse the gels into a dried buckypaper, vacuum filtration was used because this approach requires the use of a high-boiling-point solvent. However, compared to the previously described process, the amount of solvent is, in this case, significantly smaller.

**Figure 2:**
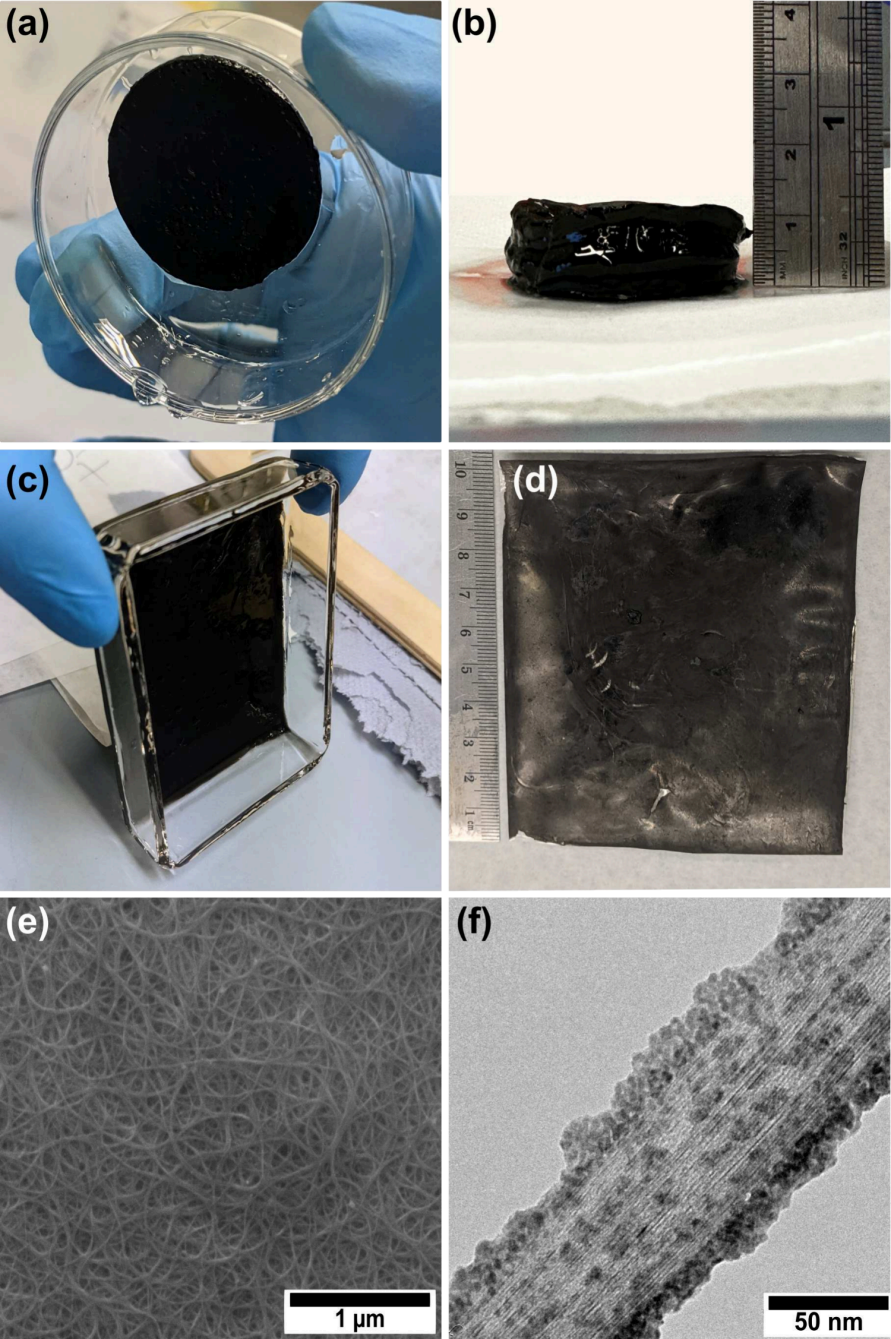
Photographs of CNT gels deposited in circular (a and b) and rectangular (c and d) Petri dishes. The dry buckypaper in part d is 10 cm × 10 cm. (e) SEM micrograph of a representative CNT mat. (f) TEM micrograph of a bundle within the CNT mat.

While the gelification process may initially appear as a disadvantage of the method, we have harnessed this effect to facilitate the deposition of large-area films of thick buckypaper. Figure 1b displays a schematic summary of the five steps involved in the developed process, and Figures S1–S3 provide supplementary photographs that document the complete process, which we describe as follows: (1) We utilize a custom 3D-printed syringe to deposit an n-type CNT-salt solution onto a porous PTFE membrane, which serves as a temporary substrate. (2) To remove any excess solvent, we pass the film over the funnel of a vacuum filtration stage. (3) The dried film undergoes a patterning process where alternating segments are selectively dedoped. To achieve this, we applied a commercial PCB lacquer on specific sections, protecting them from further processing. The exposed sections are subjected to a photolysis reaction using H_2_O_2_, which oxidizes the CNT layer, effectively dedoping it.^54−56^ (4) The patterned film is then transferred onto the desired final substrate, such as PET, while simultaneously detaching it from the provisional PTFE membrane. (5) Depending on the desired configuration of the module, the film is either rolled or folded, similar to other approaches in the literature.^18,57^ In some cases, an additional electrically insulating layer may be required. For our experiments, to improve thermal contact, we glue a thin copper layer to the ends of the module with nonelectrically conductive thermal paste, as seen in the cross-sectional representation and photograph of Figure 1c. We believe that this fabrication approach significantly overcomes the barriers to the large-scale production of CNT-based OTEG modules. For example, narrow-band Internet of Things (NB-IoT) radios consume varying amounts of power, ranging from 10 to 700 mW, depending on their mode of operation (transmitting, receiving, or idle).^58^ Based on recent demonstrations of CNT-based OTEGs,^5^ we estimate that at least 10 legs would be needed to power a NB-IoT radio in idle mode, between 100 and 300 legs for receiving mode, and potentially thousands of legs for transmitting mode. Continuous films like the one presented in Figure 1d, which is more than half a meter long, bring us closer to achieving this goal. The proposed method offers great flexibility because it not only enables the deposition of long buckypaper films but also allows for the fabrication of sheets with arbitrary shapes when they are cast into a mold, as illustrated in Figure 2a,b. This approach presents an alternative to traditional roll-to-roll processing because it allows for parallelization by utilizing multiple molds. This parallelization produces large-area sheets, as depicted in Figure 1c.

As depicted in Figure 2e,f, the resulting dried buckypaper mats consist of CNT bundles with an average width of 50 nm. These bundles are coated with potassium aggregates, which are likely in the form of potassium hydroxide based on energy-dispersive spectroscopy (EDS) measurements (refer to Figures S4 and S5). Notably, the bundle size in our buckypaper is twice as large as the bundle size observed in other sodium dodecylbenzenesulfonate- and alkali-metal-based buckypaper.^5^ However, because we intentionally allow the CNTs to aggregate in solution by exposing them to air in order to form the gel, a larger bundle size compared to that for films prepared in an inert atmosphere was expected.

### Thermoelectric Properties

We investigated various parameters that could affect the performance and scalability of the resulting films. We have observed that one critical factor is the selection of solvents employed for solubilizing the CNTs because it determines the number of steps involved in the process. For example, (i) in a conventional approach, (1) CNTs are exposed to a reductant agent dissolved in THF that negatively charges the CNTs. Then, the charged CNTs are (2) washed and (3) dried, forming a CNT salt, before (4) they are redissolved in a polar aprotic solvent like DMSO.^59,60^ Alternatively, a more recent approach (ii) requires only one step and relies on using a single solvent (i.e., DMAc) as a good solvent for both the reductant agent and the charged CNTs. Essentially, all of the materials are combined in a single pot.^60,61^ The main differences between these methods (i and ii) are the number of steps involved and the final content of alkali metal due to the extra washing step in method i. The thermoelectric properties of the films using both methods exhibit minimal variation, as illustrated in Figure S6. Nonetheless, we expect the method involving fewer steps and better control over the alkali metal, i.e., the doping level, to be a better choice for a large-scale fabrication process.

**Figure 3:**
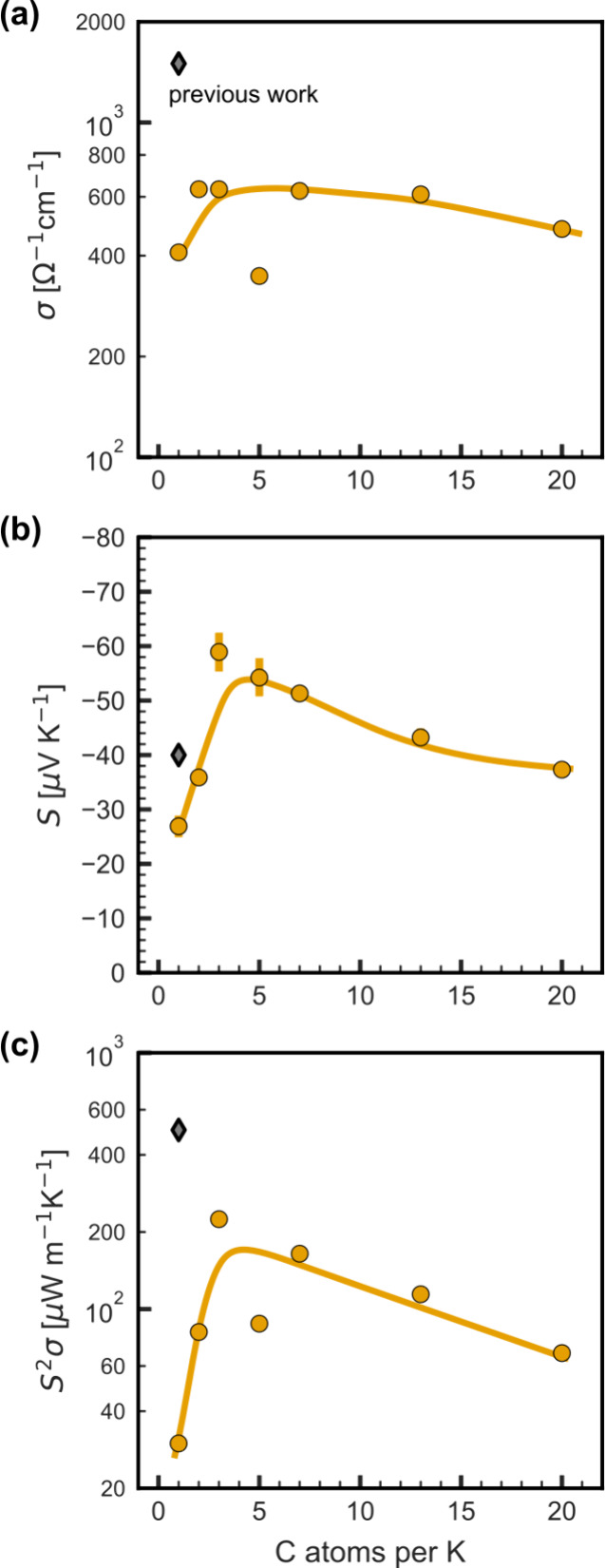
(a) Electrical conductivity, (b) Seebeck coefficient, and (c) power factor of CNT films prepared using different carbon-to-potassium molar ratios. Solid lines are a guide for the eye. The phrase “previous work” corresponds to work by Dörling et al.^5^

Next, we examined the impact of the carbon-to-potassium molar ratio on the thermoelectric properties, with the goal of achieving a high absolute Seebeck coefficient and power factor. The results are displayed in Figure 3. While the electrical conductivity remains relatively constant, the optimal Seebeck coefficient and power factor are observed at a ratio of three carbon atoms per potassium. Consequently, for all subsequent samples, we targeted this ratio or slightly lower because an excess in the dopant is expected to act as a buffer, potentially enhancing stability (see below). The films in this work show a lower electrical conductivity than other CNT-salt-based films and aligned CNT fibers fabricated through wet-spinning or vacuum filtration.^38,40,62−64^ As mentioned in the previous section, we attribute this to gelification and the resulting larger bundle size observed in this work as well as a larger porosity (vide infra).

Finally, in order to calculate the figure of merit, we measured the in-plane thermal conductivity of the CNT films with a carbon-to-potassium ratio of 3, as shown in Figure S7. For this purpose, we have used a recently developed method^44,45^ by some of the authors, which provides enhanced sensitivity to in-plane thermal transport. We obtained a thermal conductivity of 10.07 W m^–1^ K^–1^, thus giving *zT* ≈ 0.01. Noteworthily, the thermal conductivity we obtained is approximately half that observed in a previous work.^5^ We tentatively ascribe this to a larger porosity (here, 55%) in our films than what is normal for other buckypapers (between 11% and 39%).^65^ Yet, we highlight that this value is just an estimate, obtained using a method that employs the equivalent circular diameter of empty spaces in a SEM micrograph to calculate their cumulative volume (see the Supporting Information).^66^

### Stability in Air and Accelerated Aging

**Figure 4:**
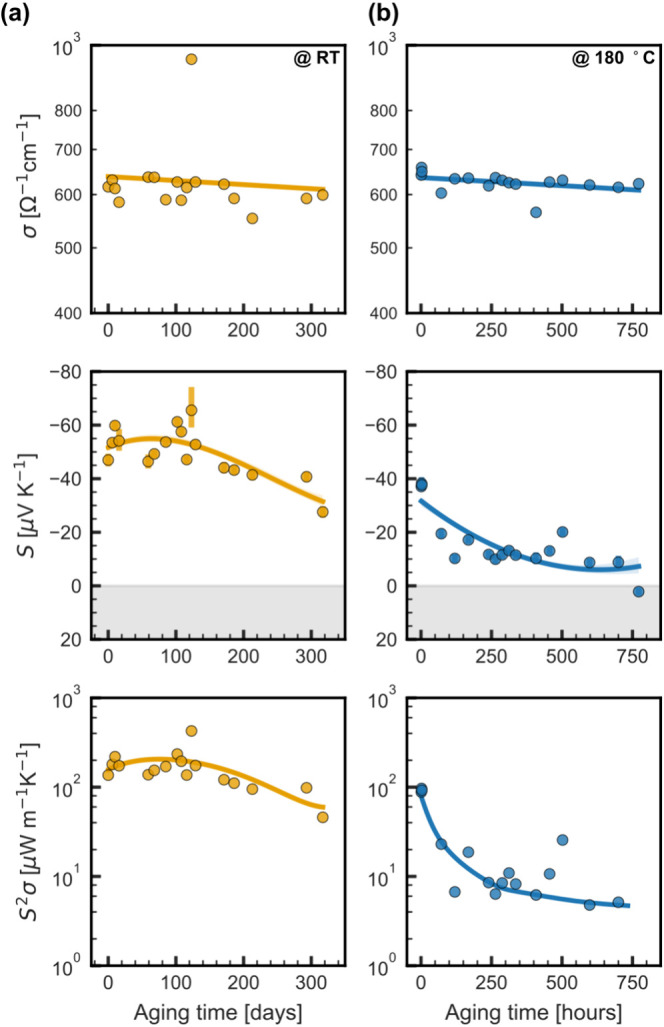
Thermoelectric performance of CNT films aged under air (a) at room temperature and (b) at 180 °C. Films were kept under these conditions and only moved for characterization. Solid lines are a guide to the eye. Shadow areas mark the change from negative to positive Seebeck coefficient.

To assess the long-term stability of our samples, we examined their performances over time under two different conditions: in air at room temperature and at 180 °C. Figure 4 illustrates the results. Over the course of 300 days at room temperature and 750 h at 180 °C, we observed minimal changes in the electrical conductivity (σ). It decreased only by 10–15% compared to the initial value, indicating that approximately 80% of the initial value would be maintained even after 400 h. By contrast, the behavior of the Seebeck coefficient (*S*) differed between the two conditions. At room temperature, *S* initially became slightly more negative before steadily decreasing to around −28 μV K^–1^ after 300 days. On the other hand, at 180 °C, *S* experienced an accelerated decrease within the first 50 h before somewhat stabilizing between −10 and −15 μV K^–1^, until eventually turning positive after 750 h. Noteworthily, these single-leg samples were not encapsulated, and unlike other works, we did not employ any crown ether to stabilize the dopant.^5,6,59,62^ Consequently, further room for improvements in the stability can be expected.

### n-to-p Patterning

As discussed above, thermoelectric generators require both p- and n-type legs. Inspired by previous works in which a p-type CNT composite is dedoped using UV radiation in order to convert it to an n-type material,^51^ we investigated whether a postprocessing step could transform the n-type CNTs into p-type CNTs. This would allow us to go from a large coated sheet of n-type buckypaper to a patterned strip of alternating n- and p-type segments to construct an OTEG. Oxidized CNTs exhibit a pronounced p-type character,^67^ which can be used to counteract the n-doping effect of the potassium metal on the CNT bundle surface. To achieve this, we drew inspiration from various photodegradation processes commonly employed to oxidize or degrade organic matter through OH^•^ radical oxidation.^68−70^ When applied to small molecules, these processes result in their fragmentation. However, in the case of larger molecules like CNTs, they lead to the generation of various oxygen-containing functional groups on the surface.^53,69,71,72^ As a source of OH^•^ radicals, we employed two methodologies: (1) H_2_O_2_ + UV and (2) UV ozone (using a commercial UVO cleaner). These two methods are quite common and are suitable for large-scale processes.^68^ For comparison, we also exposed the CNT films to plain H_2_O_2_ or UV light to determine whether oxidation or dedoping could occur in an even simpler fashion. We studied the effect of these postdeposition treatments on films prepared with multistep (with DMSO) and single-step (with DMAc) techniques for different time intervals.

**Figure 5:**
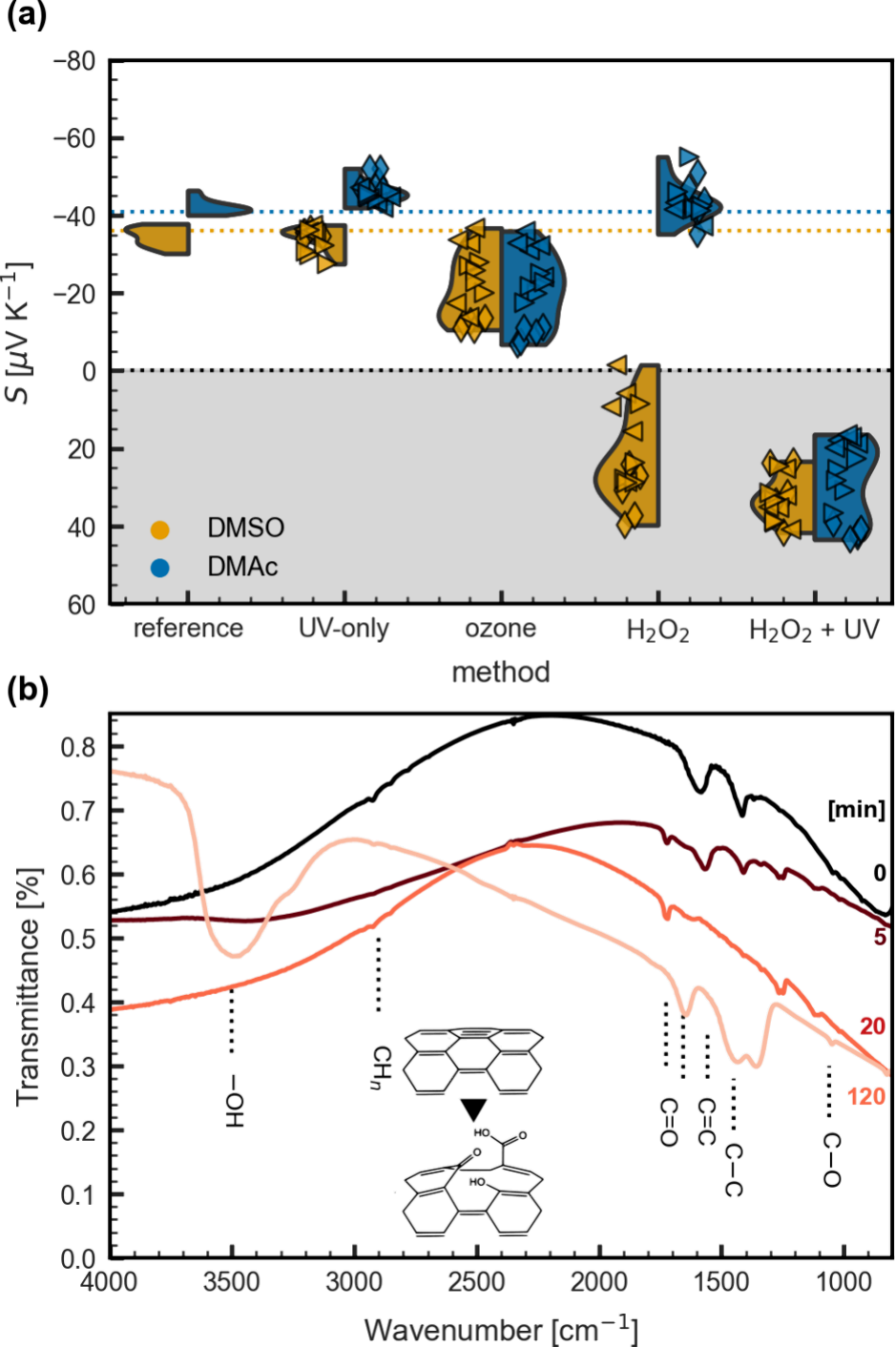
Oxidation of CNT films. (a) Seebeck coefficient of representative samples dedoped using different oxidation methods and time intervals, ranging from 5 min (left pyramid) and 10 min (right pyramid) to 20 min (diamond). (b) FTIR spectra of a CNT film treated with H_2_O_2_ + UV for different time intervals. Measurements were taken on a free-standing film. Shaded areas show the distribution density for each method. We measured samples per time condition.

As can be seen in Figure 5a, the conversion efficiency from n to p type, as seen in the resulting Seebeck coefficient, strongly depends on the method and type of sample. UV-only does not seem to have any effect, but the ozone reference does produce some degree of oxidation in the absolute value of the Seebeck coefficient, albeit it is not entirely reproducible. Treating the samples with H_2_O_2_ has a different effect depending on the type of sample, totally changing the electronic character for samples prepared from DMSO while leaving those from DMAc unaffected. We attribute this to the fact that the additional washing step during the preparation of the DMSO samples removes excess dopant. Therefore, samples prepared from different solvents with nominally the same amount of dopant probably do, in fact, differ, which in this case manifests as decreased stability toward degradation. The H_2_O_2_ + UV treatment appears to be the most efficient in turning the Seebeck coefficient positive because it works from both types of samples, namely, prepared from DMSO and DMAc. For the latter, we note that the H_2_O_2_ + UV treatment had to last longer than at least 20 min for complete transformation. The H_2_O_2_ + UV treatment with IR spectroscopy showcased in Figure 5b indicates the formation of various C=O groups as a result of the oxidation process, agreeing with previous publications.^56,69^ For the next set of experiments, and thinking about stability and scalability, we selected DMAc as a solvent and the H_2_O_2_ + UV treatment for the patterning of legs.

In order to validate the effectiveness of the developed methods, we proceeded to fabricate two prototypes of thermoelectric generators and conducted a comprehensive performance analysis. We manufactured two thermoelectric generator prototypes: a 4-leg module and a 10-leg module. The n legs were fabricated as deposited, while the p legs underwent a treatment involving H_2_O_2_ plus UV irradiation. To prevent compression of the modules during characterization, we utilized cork supports to sandwich the modules. Additionally, to enhance heat transfer onto the thermoelectric legs, we attached a copper sheet to the sides of the module using a nonelectrically conductive thermal paste, as depicted in Figure 1c.

**Figure 6:**
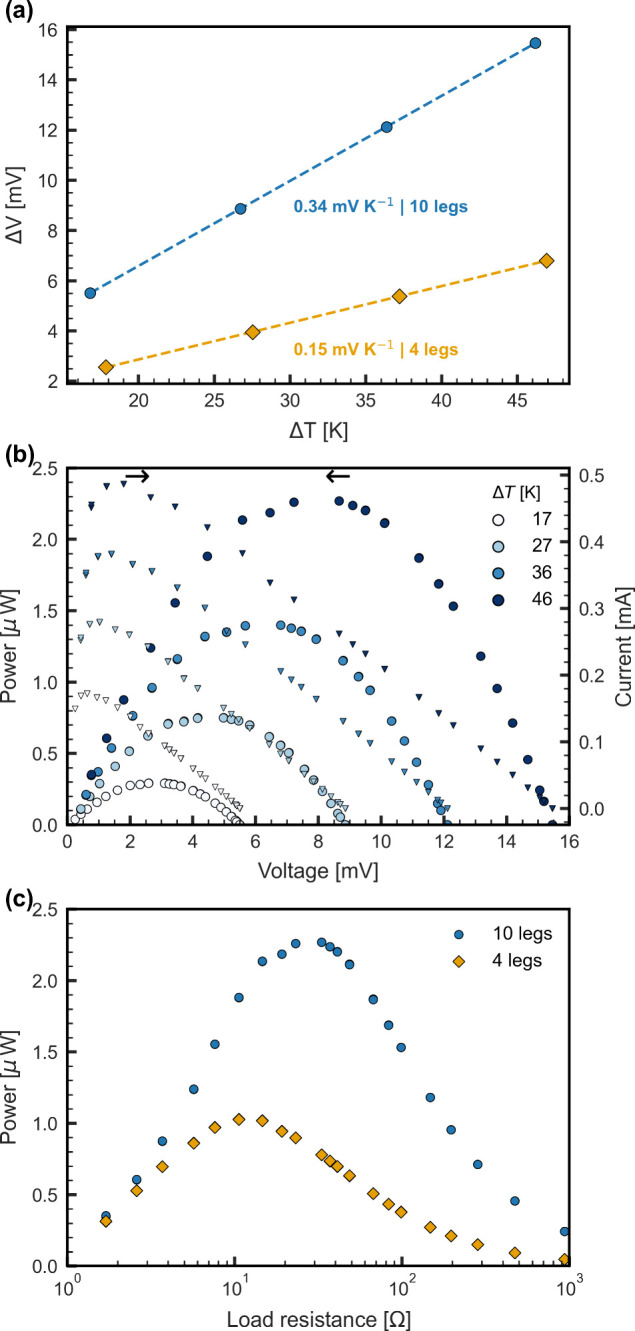
Thermoelectric characterization of the OTEG modules. (a) Seebeck coefficient of the fabricated modules. (b) Current and power of the 10-leg module at different hot-side temperatures. (c) Power versus load resistance of both modules at a Δ*T* value of 46 °C.

The modules were characterized by placing them between an aluminum heater and a heat sink. The temperature of the hot side was stabilized at temperatures of 40, 50, 60, and 70 °C, resulting in Δ*T* values of 17, 27, 36, and 46 °C, respectively. The total Seebeck voltage was 0.34 and 0.15 mV K^–1^ for the 10-leg and 4-leg modules, respectively, as seen in Figure 6a. These values correspond to a Seebeck voltage of about 34–37 μV K^–1^ per leg, which is somewhat lower than the theoretically expected values of 50 and 40 μV K^–1^ for n- and p-type legs, respectively. We attribute these differences to imperfectly dedoped legs and uneven thermal contact between the thin thermoelectric components and the copper contacts.

Figure 6b shows the power output characteristics of the 10-leg module for different Δ*T* values, and Figure 6c summarizes the power output of both modules at a Δ*T* value of 46 °C as a function of the load resistance.

## Conclusions

We have introduced a methodology for creating OTEGs using a single-component ink. Our approach involves depositing a thick film of CNT gel and then locally dedoping the homogeneous n-type CNT sheet, resulting in alternating p-type and n-type segments. These segments can be folded together to form a fully interconnected module without the need for additional metallic interconnections.

The presented method offers several advantages that are applicable to large-scale processes, such as roll-to-roll and screen-printing techniques. Additionally, the resulting n-type legs exhibit remarkable stability in air, even without any encapsulation. At room temperature, they retain 80% of their power factor after 100 days and up to 30% after 300 days. Under accelerated aging conditions at 180 °C in air, the power factor drops to 10% of the initial value within the first 10 h and further decreases to 8% after 700 h.

## Supporting Information Available

Data and figures including representative photographs of the fabrication process and thermal conductivity, TEM and SEM micrographs, EDS, and estimation of the porosity of the CNT films (PDF)
