# Supplementary material for: Upscaling Thermoelectrics: Micron-Thick, Half-a-Meter-Long Carbon Nanotube Films with Monolithic Integration of p- and n-Legs
Source: ACS Appl Electron Mater. 2024 Mar 5;6(5):2978–87. doi: 10.1021/acsaelm.3c01671 (PMC11137818; doi:10.1021/acsaelm.3c01671)
Supplement: Supplementary file 1 — el3c01671_si_001.pdf [file el3c01671_si_001.pdf]

Supporting Information: Upscaling thermoelectrics: Micron-thick, half-a-meter-long carbon nanotube films with monolithic integration of p- and n- legs

Osnat Zapata-Arteaga,† Bernhard Dörling,† Ivan Alvarez-Corzo,† Kai Xu,† Juan Sebastián Reparaz,† and Mariano Campoy-Quiles*,†,‡

† *Instituto de Ciencia de Materiales de Barcelona (ICMAB-CSIC), Bellaterra, 01893 Spain*

‡*corresponding*

E-mail: mcampoy@icmab.es,m.campoy@csic.es

Contents

1 Fabrication process **S-2**

2 Scanning Electron Microscopy **S-3**

3 Thermoelectric properties of CNT-salts from different solvents **S-4**

4 Frequency domain thermoereflectance **S-5**

5 Porosity estimation **S-8**

References **S-9**

1 Fabrication process

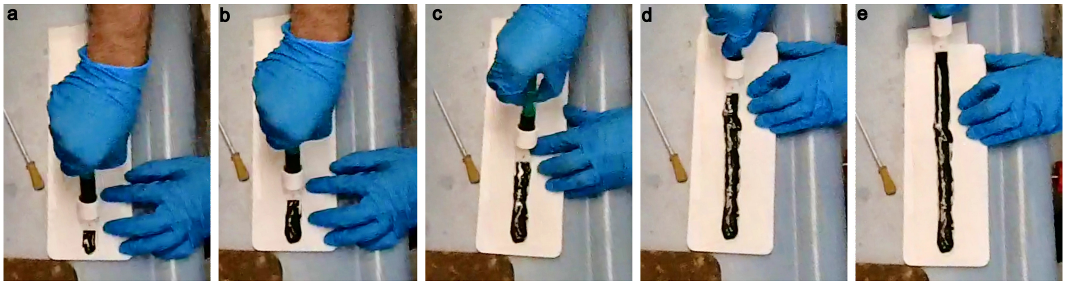

Figure S1: Photographs of the deposition process for fabricating a continuous film of the CNT-salt ink.

Large area films of approximately 1 cm x 20 cm were fabricated by depositing 20 ml of the CNT solution onto a porous polytetrafluoroethylene (PTFE) filter sheet using a syringe coupled to a 3D-printed slot-die as seen in Figure S1. The CNT ink formed a gel upon contact with air which was dried by sliding the porous PTFE membrane through a vacuum stage, as seen in Figure S2. As a first embodiment of our experiments, the drying stage was a Büchner funnel filtration setup.

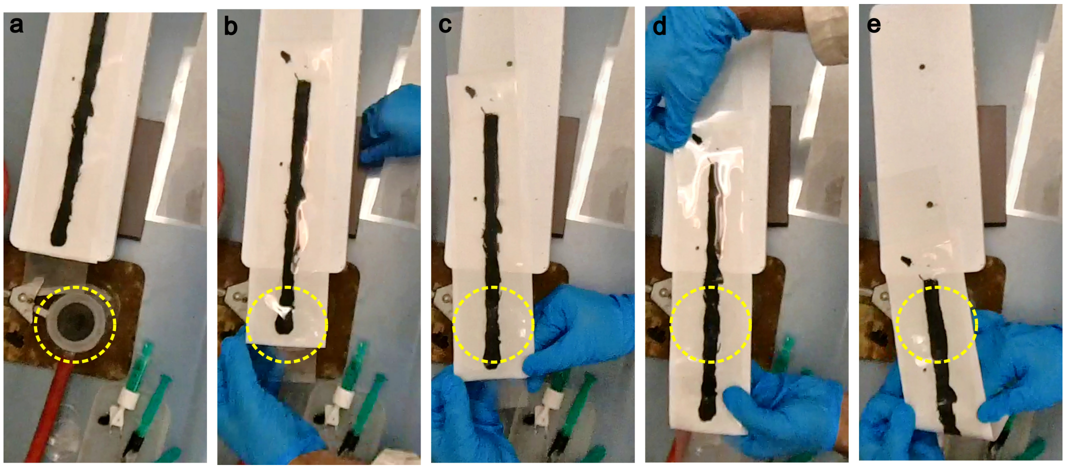

Figure S2: Photographs of the drying process. The yellow ring indicates the position of the Büchner funnel filtration setup.

The proposed method allows to transfer of the large-area film from the temporary PTFE substrate and onto a polyethylene terephthalate target substrate by making a good conformal contact and allowing to fully dry the CNTs in the new substrate. A representative process

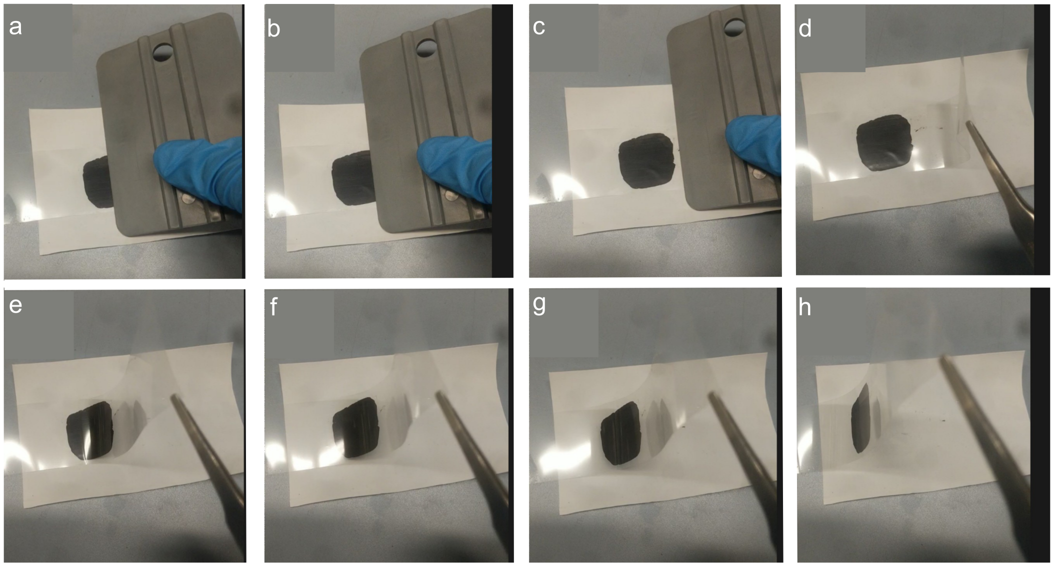

Figure S3: Photographs of the substrate transfer process. Pictures were taken sequentially, i.e., from (a) to (h). Large-area CNT films can be transferred easily to a target substrate by making conformal contact between the CNT film and the target substrate and allowing the film to dry. After that the films transfer automatically to the target substrate. Numbers indicate the sequence of photographs taken during the substrate transfer process

of the substrate transfer process is shown in FigureS3.

2 Scanning Electron Microscopy

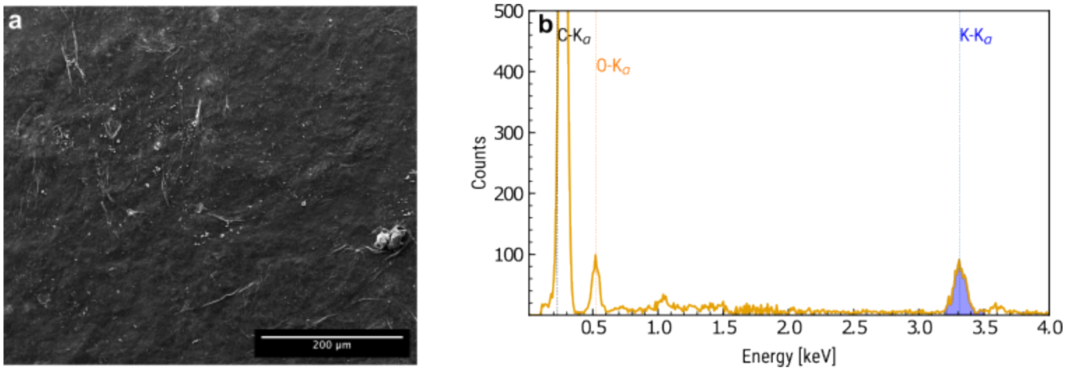

Figure S4: (a) Scanning electron micrograph and (b) Energy dispersive spectroscopy (EDS) of dry CNT films.

SEM micrographs show various patches of a white residue which EDS indicate are composed primarily of potassium.

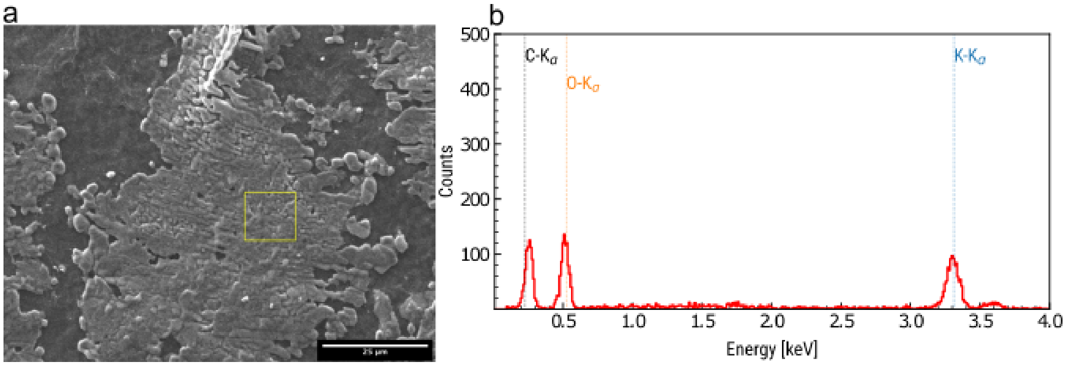

Figure S5: (a) SEM and (b) EDS of a dry CNT mat. Accumulated dopant is clearly visible on the surface.

3 Thermoelectric properties of CNT-salts from different solvents

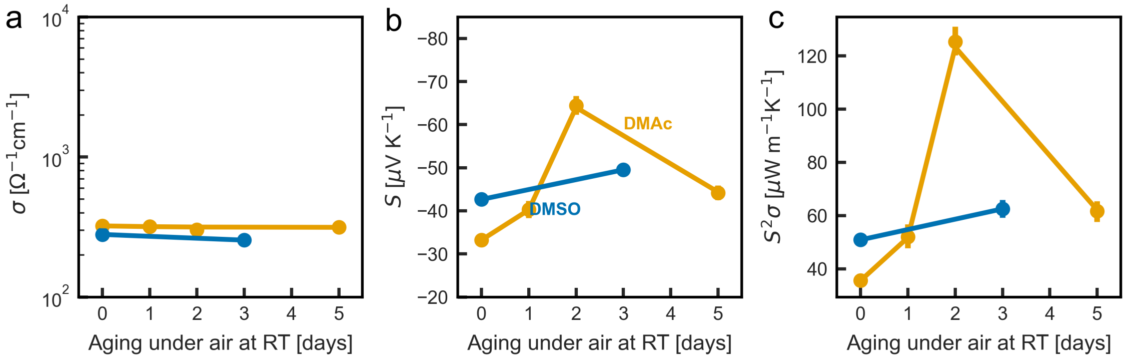

Figure S6: (a) Seebeck coefficient, (b) Electrical conductivity and (c) power factor for carbon nanotubes prepared using a multi-step route (with DMSO), and a one-step route (with DMAc). Both materials were prepared from identical C-K ratios.

Over the short term, the thermoelectric properties of CNT films prepared using the different methods do not vary significantly. Yet, it is expected that the total metal alkali content is higher in samples prepared using the single-step methodology. For high dopant content, this excess is visible in micrographs and by eye as large patches of a white residue on the surface of the film, as seen in Figure S5.

4 Frequency domain thermoereflectance

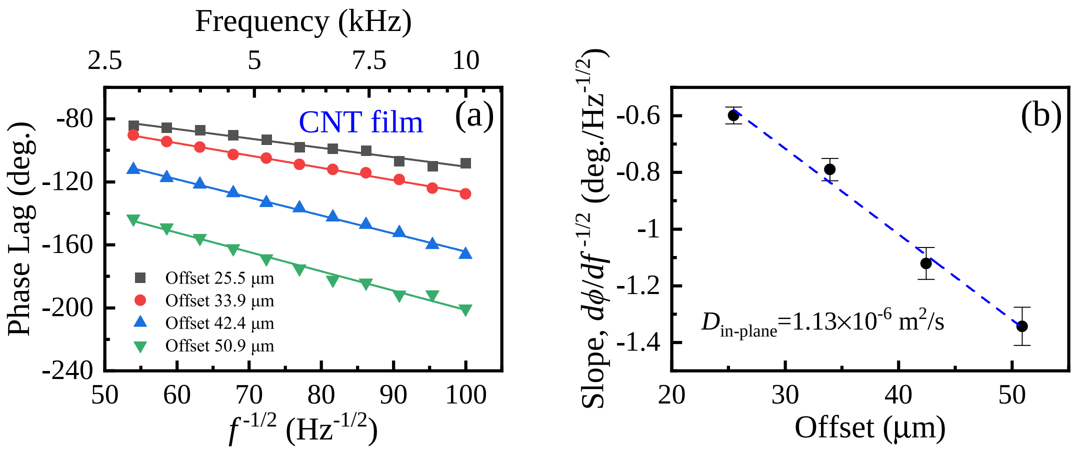

Figure S7: Representative measurements for the Frequency domain thermoreflectance experiments. (a) phase lag as a function of the heating frequency. (b) Fit of the phase lag as a function of the spatial offset.

In order to measure the in plane component of the thermal conductivity we used a novel method based on frequency domain thermoreflectance with an ofset between pump and probe. As shown in Figure S7a, we chose four spatial offset values in the range of 25.5 to 50.9 μm and measured the phase lag between the line sharped pump and probe versus heating frequency in each case. The average incident power of the pump laser is around 40 mW and the and the modulation frequency range is approximately 2.5 to 10 kHz. The slope of the linear fit of phase lag versus square root of frequency ($d\phi/df^{1/2}$) is plotted against the spatial offset (r) as in Figure S7b. The in-plane thermal diffusivity (D) of the CNT film is thus determined by a linear fit of the data points in Figure. S7b according to the equation:

$$\frac{\partial^2\phi}{(\partial r)(\partial f^{1/2})} = \sqrt{\frac{\pi}{D}} \tag{S1}$$

The thermal conductivity was calculated using:

$$\alpha = \frac{\kappa}{c_p * \rho} \tag{S2}$$

Where α is the thermal diffusivity, κ is the thermal conductivity, c_p is the heat capacity, and ρ is the density. c_p and ρ were taken from the literature.[1,2] The resulting thermal conductivity for our films was $10.7\,\mathrm{W\,m^{-1}\,K^{-1}}$.

It is important to note that we do not expect a strong dependence on the thickness of the sample, as can be deduced by the linear response observed in Figure S7a where the phase lag between the thermal excitation and the thermal response of the sample is plotted as a function of $\partial f^{1/2}$. For all excitation frequencies the response is linear (i.e., Phase Lag vs. $\partial f^{1/2}$) as predicted by the expression for the thermal penetration depth. This implies that the system must have a single value for the thermal conductivity since, in the opposite case, a non-linear response would be expected. In addition, Figure S7b also exhibits a linear response of the Phase Lag as a function of the spatial offset, hence, confirming the previous conclusion.

The extent of the spatial region probed by this method is determined solely by the thermal properties of the sample and by the modulation frequency used to drive the heat source (defined by the pump laser). In fact, the thermal penetration depth is expressed as:

$$\mu = \sqrt{\frac{D}{\pi f}} \tag{S3}$$

Hence, we using $D = 1.13 \cdot 10^{-6} m^2/s$ (see Figure S7), and the minimum and maximum excitation frequencies used in the experiments, $f_{min} = 2.5$ kHz and $f_{max} = 10$ kHz, we obtain the minimum and maximum thermal penetration depths as: $\mu_{min} = 5.5\mu m$ and $\mu_{max} = 12\mu m$. Hence, for all measurement conditions the thermal penetration depth is always much larger than the thickness of the samples ($< 1\mu m$). Hence, the entire sample thickness is probed independently of the used excitation frequency. In other words, the present method allows to study samples with low thermal conductivity using rather large thermal penetration depths, which originates from the rather low frequency range used in the measurements. This can be easily verified considering that signal is still observable at spatial offsets (between the heater and thermometer) as large as 50 μm as shown in Figure

S7.

Finally, we note that the thermal penetration depth must not be confused with the optical penetration depth of the lasers on the sample, which can be effectively much smaller than the thermal penetration depth. The later quantity is determined by the optical absorption coefficient of the CNT films at the excitation wavelength (405 nm), and it has no direct influence on the thermal response at distances sufficiently large distances the heat source, typically $> 5\mu m$. We have used a lowest spatial offset of around $25\mu m$, hence fulfilling the previous condition.

5 Porosity estimation

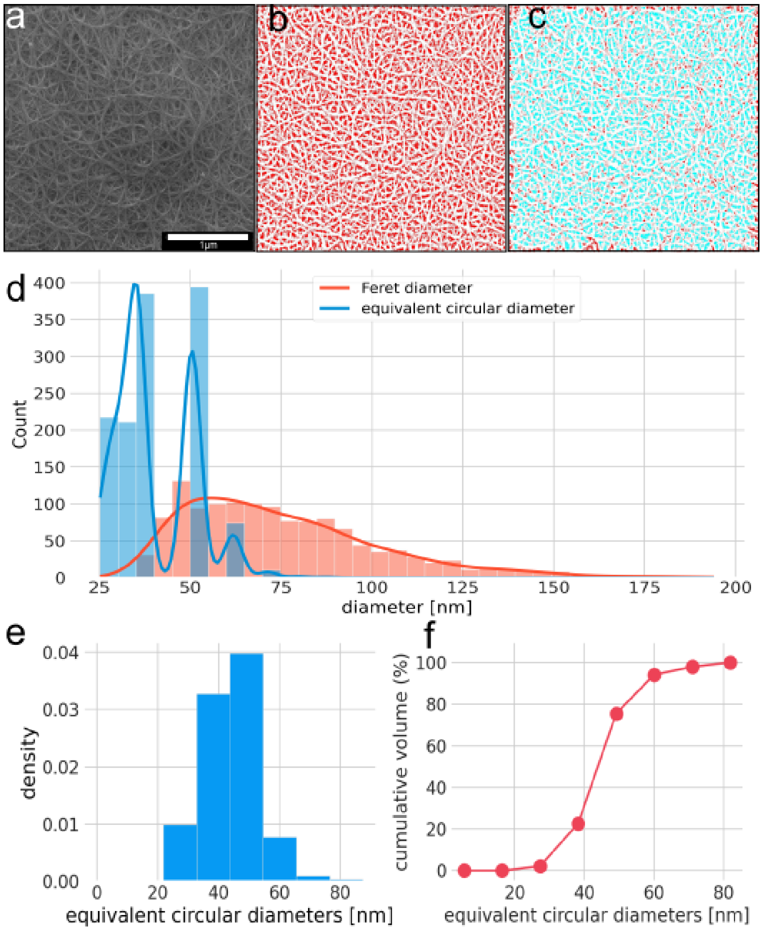

Figure S8: Binarization of an SEM image. (a) Original SEM image, (b) binarized SEM image (c) Exclusion of incomplete 'pore' on the edge of the image. (d) Calculation of the pore Feret diamenter and equivalent circular diameter. (e) Estimation of the density of various pores and (f) their cumulative volume.

The Saltykov method is particularly suitable for estimating the volume percentage of a given grain-size distribution taken from microscopy images.[3] For this work, we make the assumption that empty spaces in a SEM image are equivalent to a 'pore' that can be defined with an equivalent circular diameter (ECD).

$$ECD = 2 \cdot \sqrt{areas/\pi} \tag{S4}$$

This can give different pore distributions for a given ECD, which then we use to estimate

the cumulative volume of the pore with the highest frequency. All analysis was done with the Fiji ImageJ software.

References

(1) Cao X., J.; Yan H., X.; Xiao, Y. Specific heat of Single-Walled Carbon Nanotubes: A Lattice Dynamics Study. *Journal of the Physical Society of Japan* **2003**, *72*, 2256–2259.

(2) Zhang, L.; Zhang, G.; Liu, C.; Fan, S. High-Density Carbon Nanotube Buckypapers with Superior Transport and Mechanical Properties. *Nano Letters* **2012**, *12*, 4848–4852.

(3) Lopez-Sanchez, M. A.; Llana-Fúnez, S. An extension of the Saltykov method to quantify 3D grain size distributions in mylonites. *Journal of Structural Geology* **2016**, *93*, 149–161.
